# Limited β_2_-adrenoceptor haplotypes display different agonist mediated airway responses in asthmatics

**DOI:** 10.1186/1465-9921-7-19

**Published:** 2006-01-31

**Authors:** Anneke van Veen, Eddy A Wierenga, Robert Westland, Frank R Weller, Guus AM Hart, Henk M Jansen, René E Jonkers

**Affiliations:** 1 Department of Pulmonology, Academic Medical Centre, University of Amsterdam, Amsterdam, The Netherlands; 2 Department of Cell Biology and Histology, Academic Medical Centre, University of Amsterdam, Amsterdam, The Netherlands; 3 Department of Epidemiology and Biostatistics, Academic Medical Centre, University of Amsterdam, Amsterdam, The Netherlands

## Abstract

**Background:**

*In vitro *and some *in vivo *studies suggested that genetic haplotypes may have an impact on β_2_-agonist mediated airway responses in asthmatics. Due to strong linkage disequilibrium the single nucleotide polymorphisms (SNPs) in the β_2_-adrenoceptor gene result in only a limited number of haplotypes. We intended to evaluate the impact of β_2_-adrenoceptor haplotypes on β_2_-agonist mediated airway responses and the development of tolerance in mild to moderate asthmatics.

**Methods:**

Patients were genotyped for the part of the β_2_-adrenoceptor gene with a known bearing on receptor function and regulation. Cumulative dose response curves of fenoterol versus PD_20 _methacholine and FEV_1 _were constructed after 2 week treatment periods with either terbutaline or placebo in a double blind, randomised and cross-over design. Analysis of the dose response curves was based on a repeated measurement analysis of covariance.

**Results:**

In our study population comprising 45 asthmatic patients, we found three limited allelic haplotypes, resulting in six different genotypes. Our data support the existence of differences between these six genotypes both in the shape of the dose response relationship of the β_2_-adrenoceptor agonist fenoterol as well as in the propensity to develop tolerance for these effects by pre-treatment with terbutaline. However, this could only be substantiated for the endpoint PD_20 _methacholine.

**Conclusion:**

Between β_2_-adrenoceptor genotypes differences exist both in baseline β_2_-agonist induced airway responses as well as in the propensity to develop tolerance during maintenance β_2_-agonist therapy. The net differences after two weeks of therapy are, however, of magnitudes that are unlikely to be of clinical significance.

## Background

Over the past decade an increasing number of single nucleotide polymorphisms (SNPs) in the β_2_-adrenoceptor (β_2_-AR) gene have been identified. Initially the focus of research was on two highly prevalent non-synonymous SNPs in the coding region of the gene that both result in an amino acid substitution in the extra-cellular part of the receptor protein: position 16 Arg→Gly and position 27 Gln→Glu. *In vitro *these amino acid changes appeared to alter the susceptibility to receptor downregulation by exposure to β_2_-agonists [1,2]. These observations fuelled a number of clinical and *in vitro/ex vivo *studies yielding inconsistent and sometimes conflicting results. Arg-16 was found to be associated with a greater acute bronchodilator response to a β_2_-AR agonist [3,4], but also with loss of asthma control in some studies [5,6], but not in all [7]. In an *ex vivo *study using human peripheral blood lymphocytes no impact of either polymorphism could be substantiated on baseline receptor expression or responsiveness[8].

It is generally assumed that the *in vivo *consequence of the downregulation of β_2_-ARs is tolerance towards the airway smooth muscle mediated effects of β_2 _agonists. This tolerance has generally been difficult to show for the bronchodilator effects of β_2_-AR agonists, but is more pronounced and potentially clinically relevant for their bronchoprotective effects [9]. The relationship between the polymorphic amino acids 16 and 27 and the susceptibility to bronchodilator tolerance was the subject of two clinical studies [10,11], but in only one of these such an association could be substantiated [10]. Tolerance development towards the bronchoprotective effects of β_2_-agonists was the subject of two prospective clinical studies, which did not find differences between amino acid 16 genotypes [12,13]. Results of *in vitro *studies using either human mast cells or airway smooth muscle cells did not aid in settling the issue [14,15].

More recently additional SNPs in the non-coding regulatory part of the β_2_-AR gene were described, some of which affect receptor expression and regulation *in vitro *[16-18]. These SNPs are in strong linkage disequilibrium with those coding for amino acid 27 in the β_2_-AR protein, which results in only a very limited variation in extended allelic haplotypes [16,18,19]. *In vitro *studies initially focused on the SNPs in the 5' flanking region of the receptor coding block in isolation. Analysis of the relative promoter activities of serially truncated fragments of the 5' flanking region suggested that the regulatory activity of the β_2_-AR gene is largely concentrated in the region of 550 base pair 5' to the coding block. In particular, deletion of the region containing the -367 SNP strongly reduced transcription. In a comparative assay, alleles containing the -367 T→ C mutation were shown to result in a lower transcription rate (~17%) [18]. Recently, we were able to confirm this finding and showed that this was associated with the decreased binding of an as yet unidentified transcription factor [20].

The intronless coding region of the β_2_-AR protein is preceded by a small open reading frame encoding a 19 amino acid peptide, the β_2_-AR upstream peptide (BUP), which inhibits β_2_-AR mRNA translation [17]. The -47 C/T SNP leads to a Cys → Arg substitution at position 19 of the BUP. Transfection experiments with constructs containing either variant of this SNP showed that Cys19 resulted in an increase in receptor protein expression through an effect on mRNA translation [16]. However, when the BUP SNP was studied in the context of a validated haplotype, the BUP Cys19 allele was associated with decreased receptor protein and mRNA expression, which appeared to be associated with a decreased bronchodilator response to an inhaled β_2_-agonist in a cross-sectional study in a cohort of asthmatics [19]. On the basis of this latter observation these authors advocated studying the biological phenotypic consequences of the β_2_-AR SNPs only within the context of validated haplotypes. In fact, our study extends on this study. For our functional analyses we limited the haplotypes to the SNPs in the 5'region of the gene, of which an influence on transcription and regulation may be expected, combined with the two far most prevalent non-synonymous SNPs in the receptor protein coding block at +46 and +79.

Our primary aim was to study the impact of different combinations of allelic haplotypes on tolerance to β_2_-agonist induced bronchoprotection. To this end, we conducted a double blind cross-over study of two-week treatment periods with either the short-acting β_2_-agonist terbutaline or a matching placebo. Cumulative dose response curves of the full β_2_-AR agonist fenoterol versus PD_20 _methacholine were used as the main physiologic endpoint.

We found differences between six distinct β_2_-AR allelic genotypes in the shape of the dose response relationship and in the propensity to develop tolerance for these effects. These differences are statistically significant and functionally relevant only for bronchoprotection when compared to bronchodilation in terms of recovery from metacholine induced bronchoconstriction. The magnitudes of the net differences are, however, unlikely to be of clinical significance

## Methods

### Patients

Recruitment of patients with mild to moderate asthma and inclusion criteria have been described in detail elsewhere [21]. According to current guidelines all patients used inhaled corticosteroids, of which the dose was kept stable from at least 8 weeks prior to inclusion until the end of the study. If inclusion criteria were met, a blood sample was drawn for isolation of DNA. All subjects gave written informed consent to participate in the study that was approved by the Medical Ethics Committee of the Academic Medical Centre in Amsterdam.

### Design

The study had a randomized, placebo-controlled, double-blind, cross-over design. Two treatment periods of two weeks were preceded and separated by wash-out periods of two weeks, during which all β_2_-agonists were discontinued and only ipratropium bromide pressurized metered dose inhaler (pMDI) was allowed for symptom relieve. During the treatment periods a dry powder inhaler (Turbuhaler^®^, Astra-Zeneca, Zoetermeer, the Netherlands) containing either 500 μg of terbutaline per inhalation or placebo was used four times daily. The subjects attended to the laboratory 24 hours after the last dose of study medication and after ipratropium bromide had been withheld for at least 8 hours. After baseline FEV_1 _and PD_20 _methacholine had been determined, subjects inhaled 200 μg of fenoterol pMDI from an aerochamber as the first of a series of 4 doubling doses, resulting in cumulative doses of 200, 600, 1400, and 3000 μg respectively. One hour after each dose of fenoterol a PD_20 _methacholine was determined, immediately after which the next dose of fenoterol was inhaled. Lung function measurements and methacholine provocation tests were done as described previously [21].

### Assessment of extended β_2_-adrenoceptor genotypes

Genomic DNA was extracted from peripheral blood mononuclear cells. Using allele-specific primers distinguishing between the -367T- and -367C-alleles, DNA was amplified by PCR, applying standard conditions. The fragment between nucleotides -367 and + 377 was amplified using sense primers 7 or 8 (Table 1) and anti-sense primer 2, and the fragment between -367 and -1081 was amplified with anti-sense primers 215 or 216 and sense primer 214. The PCR products were separated by agarose gel electrophoresis and isolated from the gel. Using the same -367 haplotype-specific primer sets, the sequence of the PCR products was determined by automatic sequencing. In case of -367 homozygous patients within the cohort studied, heterozygous polymorphisms downstream or upstream were limited to the +46 SNP, thus still allowing for the assessment of the full haplotypes.

**Table 1:** Primers used for allele-specific PCR amplification and sequencing.

Primer	Fragment	direction	Sequence
2	+396/+377	Antisense	5'-gtagcgatccactgcgatca-3'
7	-387/-367	Sense	5'-gggccccgcccgggccagcc**t**-3'
8	-387/-367	Sence	5'-gggccccgcccgggccagcc**c**-3'
214	-1081/-1061	Antisense	5'-ctgcaaattcctaaggagggc-3'
215	-349/-367	Antisense	5'-ctcgccctccttctcctg**a**-3'
216	-349/-367	Antisense	5'-ctcgccctccttctcctg**g**-3'

### Statistical analysis

Patients were divided into subgroups according to their established allelic genotypes, based on combinations of the three found limited allelic haplotypes I, II, and III. FEV_1 _values are presented as % predicted, methacholine provocation test results as (geometric mean) PD_20 _(μg). Baseline FEV_1 _and PD_20 _are those measured after a two week wash out period followed by a two week placebo treatment period and before administration of the first dose of fenoterol.

Because of the markedly skewed distribution of PD_20 _values, these were logarithmically transformed prior to analysis. Analysis of the dose response curves was based on a repeated measurement analysis of covariance with log(PD_20_) or FEV_1 _(% predicted) as dependent variable, fenoterol dose, treatment (terbutaline vs. placebo), combined allelic genotype and period as factors, baseline log(PD_20_) or FEV_1 _respectively as a covariate and patient as subject within whom repeated measurements may be correlated. An unstructured covariance matrix was used, implying possible differences in SD's at the 8 different fenoterol doses by treatment combinations, as well as varying within-patient correlations between these 8 measurements (heteroscedastic). In the model, all possible interactions were allowed between the three factors fenoterol dose, treatment (terbutaline vs. placebo) and combined allelic genotype. P-values were calculated from a Wald-based F-test with denominator degrees of freedom from the "within-between" method. The analysis comprised a total of 8 global model and 6 within-genotype comparisons and associated P-values. Standard, P-values were not adjusted for multiple comparisons.

For PD_20 _results (means and means ± SE's) from the analyses were back-transformed to the normal scale. The statistical package SAS 8.2 was used for the calculations.

## Results

### Patient characteristics

A total of 50 patients were enrolled of whom 45 (11 male/ 34 female), with a mean FEV_1 _of 84.6 % predicted, and a geometric mean PD_20 _methacholine of 163 μg completed the study. Two patients discontinued the study because of side effects of the study medication (palpitations and tremor). Two patients were excluded because of not allowed use of β_2_-AR agonist as was one female patient that turned out to be pregnant during the course of the study.

The cohort of the 45 patients that completed the study comprised 5 haplotypes within the part of the β_2_-AR gene between nucleotides -1023 and + 79 (table 2), two of which occurred only single, one of which (Ic) has not been described previously. As described by others, linkage disequilibrium was found between nucleotides -367 T/C, -47 T/C (coding for Arg/Cys19 of the 5' leader peptide (5'LP)), -20 T/C and + 46 A/G (coding for Glu/Gln27 of the β_2_AR protein). This resulted in the presence within the cohort of only 3 limited haplotypes with considerably differences in (relative) frequencies (table 3). Baseline patient characteristics of these combined limited allelic haplotypes (or allelic genotypes) (table 3) as well as those of the subgroups based upon the amino acid 16 and 27 polymorphisms displayed no statistically significant differences.

**Table 2:** Localization of SNPs and delineation of haplotypes of the β2-AR gene in the cohort. The limited haplotypes correspond to the SNPs in boldface. Haplotypes between brackets correspond to those of Drysdale et al.

Nucleotide:	-1023	-654	-468	**-367**	**-47**	**-20**	**46**	**79**		
Alleles	G/A	G/A	C/G	**T/C**	**T/C**	**T/C**	**G/A**	**C/G**		
Limited Haplotype									n	Frequency (%)
III Arg19Gly16Glu27 (2)	A	G	G	C	C	C	G	G	39	43,3
Ia Cys19Arg16Gln27 (4)	G	A	C	T	T	T	A	C	36	40
II Cys19 Gly16Gln27 (6)	G	A	C	T	T	T	G	C	13	14,4
Ib Cys19Arg16Gln27 (1)	A	G	C	T	T	T	A	C	1	1,1
Ic Cys19Arg16Gln27 (x)	G	G	G	T	T	T	A	C	1	1,1

**Table 3:** Frequencies and patient characteristics of the subgroups formed by the combined limited allelic hap1otypes. Baseline FEV_1 _and PD_20 _were measured after a two week washout period followed by a two week placebo treatment period, before administration of the first dose of fenoterol.

***Genotype***	Frequency	Baseline FEV_1 _(% of predicted), mean (range)	Baseline PD_20 _geometric, mean (range)	Inhalation steroid dose, mean (range)
I/III	14 (31%)	85 (59 – 107)	141 (24 – 761)	470 (200 – 1200)
III/III	10 (22%)	86 (54 – 104)	313 (92 – 1240)	440 (200 – 1000)
II/III	5 (11%)	81 (72 – 99)	110 (20 – 371)	380 (200 – 500)
I/I	10 (22%)	85 (60 – 101)	176 (29 – 672)	530 (200 – 1000)
I/II	4 (9%)	82 (70 – 94)	74 (30 – 157)	500 (400 – 800)
II/II	2 (4%)	88 (82 – 93)	148 (33 – 668)	500 (200 – 800)

### Influence of the genetic polymorphisms and of terbutaline pre-treatment on the β_2_-agonist mediated effects on asthmatic airways

#### A. Bronchoprotection

**Figure 1:**
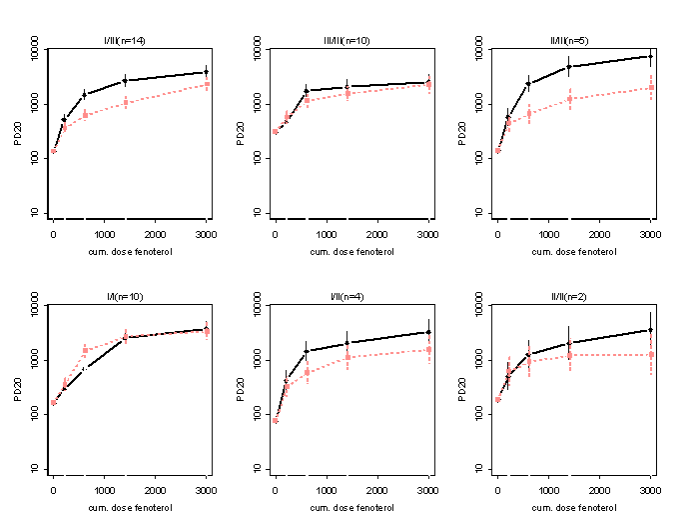
PD_20 _methacholine (mean ± SE) before and after cumulative doses of fenoterol in patients with different genotypes, pre-treated with placebo or terbutaline for two weeks. Averaged over treatment, the shape of the dose response curves varies between the genotypes (p = 0.0011). Averaged over fenoterol dose and genotype, terbutaline pre-treatment reduced PD_20 _compared to placebo pre-treatment (P = 0.0026). For the reductions in PD_20 _per genotype and associated p-values: see table 4. Drawn line: placebo pre-treatment, dashed line: terbutaline pre-treatment

Baseline PD_20_ methacholine values after placebo pre-treatment as compared to terbutaline pre-treatment were of a similar magnitude and not statistically different. Figure 1 shows the back-transformed means and SE's for PD_20 _methacholine, estimated from the model described above and corrected for baseline. There was no evidence that the shape differences on the log-scale between the dose-response curves for the two treatments vary between the allelic genotypes (interaction: pre-treatment * fenoterol dose * genotype, P = 0.23). However, there was evidence that the difference in PD_20 _between the two treatments, averaged over fenoterol dose, is related to allelic genotype (interaction: pre-treatment * genotype, P = 0.0029) and also that the shape of the dose-response curve, averaged over both treatments, varies between the allelic genotypes (interaction: fenoterol dose * genotype, P = 0.0011). No firm evidence was found that the relative difference in PD_20 _between terbutaline pre-treatment and placebo pre-treatment varies over the fenoterol dose (interaction: pre-treatment * fenoterol dose, P = 0.071), giving additional support for the averaging over fenoterol dose. Averaged over fenoterol dose and genotype, terbutaline pre-treatment reduced PD_20 _compared to placebo pre-treatment (P = 0.0026). Terbutaline pre-treatment reduced PD_20 _by 53% (95% CI: 31–68%, P = 0.0004) on average for I/III patients and by 66% (95% CI: 35–82%, P = 0.0019) for II/III patients (table 4). I/II patients showed a comparable, but non-significant reduction. For the other genetic groups, reductions are lower, if existing at all (Table 4). Adjustment for multiple comparisons – by for instance the Bonferroni correction – would not change these conclusions qualitatively. After application of a most conservative approach, i.e. by multiplying the uncorrected P-values by 14, the highest significant P-value of 0.0029 for the pre-treatment * genotype interaction would still remain below the level of 0.05, namely 0.041. For the within-genotype significant P-values for genotypes I/III and II/III the values would become 0.0056 and 0.0154 respectively.

**Table 4:** The reduction in bronchoprotection by fenoterol after terbutaline pre treatment as compared to placebo pre treatment. The response was averaged over fenoterol dose 200–3000 μg. A 50% reduction corresponds to one double dose reduction of PD_20 _methacholine. Negative numbers indicate an increase in response.

	Reduction in PD_20_			
Allelic genotype	Median	95% CI		P-value

I/III	53%	31%	68%	0.0004
III/III	22%	-24%	50%	0.30
II/III	66%	35%	82%	0.0019
I/I	-51%	-139%	4%	0.078
I/II	50%	-2%	76%	0.059
II/II	25%	-106%	73%	0.58

#### B. Recovery by fenoterol of methacholine induced bronchoconstriction

**Figure 2:**
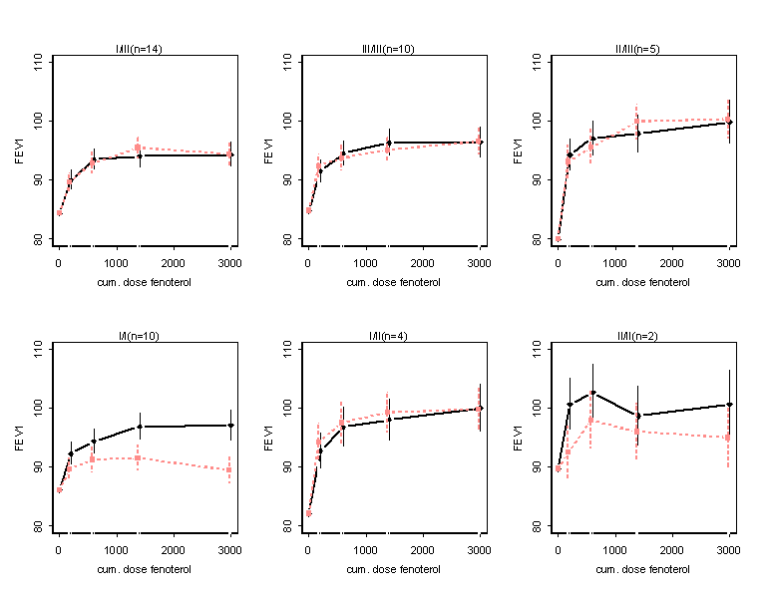
FEV_1 _(mean ± SE) before and after cumulative doses of fenoterol in patients with different genotypes, pre-treated with placebo or terbutaline for two weeks. FEV_1 _was measured one hour after fenoterol inhalation, directly after the previous PD_20 _measurement. For the reductions in FEV_1 _per genotype and associated p-values: see table 5. Drawn line: placebo pre-treatment, dashed line: terbutaline pre-treatment.

Figure 2 shows means and SE's for FEV_1 _measured one hour after fenoterol inhaled directly after the previous PD_20 _measurement, as estimated from the model and corrected for baseline. There was no evidence that the shape differences between the dose-response curves for the two treatments vary between the allelic genotypes (interaction: pre-treatment * fenoterol dose * genotype, P = 0.48). Neither was there evidence that the difference in FEV_1 _between terbutaline pre-treatment and placebo pre-treatment varied over the fenoterol dose (interaction: pre-treatment * fenoterol dose, P = 0.46), nor that the difference in FEV_1 _between the two treatments, averaged over fenoterol dose, is related to allelic genotype (interaction: pre-treatment * genotype, P = 0.29, see table 5). There was weak evidence that the shape of the dose-response curve, averaged over both treatments, varies between the allelic genotypes (interaction: fenoterol dose * genotype, P = 0.060). There was some evidence that averaged over fenoterol dose and genotype terbutaline decreases FEV_1 _compared to placebo (P = 0.027) by an estimated 1.58 (SE 0.68) percent points. Only for genotype I/I this decrease reached statistical significance (see table 5). Adjustment for multiple comparisons, by for instance the Bonferroni correction, reduced all these findings to non-significance.

**Table 5:** Decrease in FEV_1 _response to fenoterol after terbutaline pre-treatment as compared to placebo pre-treatment. Response was averaged over fenoterol dose 200–3000 μg. Negative numbers indicate an increase in response.

	Decrease in FEV_1 _(percent points)			
Allelic genotype	Mean	95% CI		P-value

I/III	0.71	-1.79	3.21	0.57
III/III	-0.28	-3.19	2.63	0.85
II/III	1.84	-2.16	5.84	0.36
I/I	3.02	0.09	5.95	0.044
I/II	-0.80	-5.28	3.68	0.72
II/II	6.04	-0.18	12.26	0.057

## Discussion

This is the first study finding differences between β_2_-AR genotypes in the shape of the dose response relationship of β_2_-AR mediated airway effects in asthmatics *in vivo*. Our data analysis, however, does not allow a further distinction in differences with respect to specific characteristics of the dose response curves, such as the maximum effect at infinite drug dose (Emax) or the dose at which 50% of this maximum effect is obtained (ED50). We found no evidence for an interaction between treatment and genotype influencing the shape of the dose response curve of PD_20 _methacholine. Neither was there evidence that the relative differences in PD_20 _after terbutaline pre-treatment and placebo pre-treatment vary over the fenoterol dose, as illustrated in figure 5. These latter two findings suggest that, at least with respect to protection against methacholine induced bronchoconstriction, there is a genotype-specific way by which binding of a β_2_-AR agonist to its receptor translates into a clinical response as well as a genotype specific but β_2_-agonist-dose-independent impact of tolerance development on this response. The latter is illustrated by the parallel course of the two dose responses curves within the different genotypes on semilogscale (figure 5).

The functional differences we observed between the allelic genotypes cannot be explained by the known functional consequences of individual SNPs or of haplotypes as delineated *in vitro*. Moreover, the functional phenotype of heterozygotic genotypes does not appear to fit in with that of the homozygotic variants. For instance, in our study genotypes I/I and III/III appear to be resistant to downregulation, while genotype I/III showed a significant degree of downregulation for bronchoprotection by fenoterol. Patients heterozygous on position 19 of the BUP and position 27 of the β_2_-AR, genotypes I/III and II/III, appeared to be most affected by desensitization, with reductions in PD_20 _of 53 and 66%, respectively. This is in line with a study that found more desensitization in human airway smooth muscle cells derived from individuals who were heterozygous on position 27 [15]. Our findings illustrate why previous studies focusing on single SNPs in the receptor protein coding block may have yielded negative or even contradictory results. For example, the sub-group of Gly-16 homozygotes consists of three genotypes, III/III, II/III and II/II, with apparently different baseline β_2_-AR agonist mediated responses and propensities to develop downregulation. This implicates that the results of functional studies based solely on variation in amino acid 16 will depend upon the distribution of genotypes within the subgroup of Gly-16 homozygotes.

The absence of a clear-cut relationship between genotypes and functional phenotypes suggests the influence of other yet unidentified co-factors. The identification of one such factor may come from recent findings in mouse models suggesting the existence of "cross-talk" in airway smooth muscle between the β_2_-AR system and G_q_-phospholipase C coupled receptors responding to contractile agonists such as methacholine [22]. The findings in this animal model of an increase in cholinergic sensitivity in the absence of chronic β_2_-AR stimulation and vice versa, fit in with our observation of a combination of apparent resistance to downregulation with respect to bronchoprotection (figure 1 and table 4) combined with the numerative (and borderline significant) largest degree of loss of bronchodilation (figure 2 and table 5) within genotype I/I. Since subjects of this genotype are homozygous Arg16/Arg16, our data in this genotype agree with those of Israel et al[6] who found an increased response to anti-cholinergic therapy in patients of this genotype when they were off β_2_-agonist therapy, in combination with no improvement in lung function when they were on β_2_-agonist therapy.

Some potential limitations of our study need to be discussed. Our active treatment arm consisted of the short acting β_2_-agonist terbutaline, where long-acting β_2_-agonists are nowadays the standard for maintenance bronchodilator therapy in asthma. What matters, however, is whether the degree of "receptor stimulation" we obtained is representative for the usual situation in maintenance therapy. In this respect it is relevant that the dose of terbutaline we employed is generally considered to be about therapeutically equivalent to the standard doses of the two long-acting β_2_-agonists formoterol and salmeterol. Furthermore, in a direct comparison 500 μg of terbutaline induced a degree of bronchoprotective subsensitivity of a same order of magnitude as the usually employed doses of formoterol [23]. Using either formoterol or salmeterol would also have limited the extent to which findings with either of this drugs can be generalized in view of their differences in intrinsic efficacy. Relevant in this respect may be that *in vitro *[24] the intrinsic efficacy of terbutaline appears to be in between those of salmeterol and formoterol. Next, the "test drug" we employed for the functional studies was fenoterol which is a full β_2_-AR agonist, like formoterol, but unlike salbutamol, salmeterol and also terbutaline that are partial agonists *in vitro *as well as *in vivo *[21,25,26]. It cannot be ruled out that the responses induced by fenoterol are stronger than those that would have been induced by a partial agonist, but it is unlikely that this would have changed the main conclusions of this study. Furthermore, the cohort we studied was of a relatively limited extent, especially in relation to the low numbers of individuals in some subgroups, particularly genotype II/II, and to the uneven presence of the different genotypes in asthmatic cohorts, as also noticed previously [19]. This implicates that the functional implications of especially genotype II/II need further study either in larger groups of patients or after pre-selection of specific genotypes.

Our genotype analyses contained all the SNPs with a known bearing on gene regulation or receptor expression, thus ignoring the three SNPs downstream from basepair +79. In our opinion this is only of limited impact. The SNPs at +252 and +523 are synonymous and so do not result in amino acid substitution, while the one at + 491 is very uncommon. However it cannot be ruled out completely that these three SNPs have an impact on receptor expression e.g. via an effect on mRNA stability.

Our study confirms that downregulation of β_2_-agonist induced airway responses is more easily substantiated for protection towards a bronchoconstrictive stimulus than for bronchodilation form "baseline", in this case: recovery form methacholine-induced bronchoconstriction one hour earlier. This is in line with a recent study showing that susceptibility to bronchodilator tolerance increases when the degree of induced bronchoconstriction increases [27]. The degree of tolerance development was much less for FEV_1 _and at the most borderline statistically significant for the cohort as a whole.

With respect to the potential clinical implications of our findings it must be realised that for all genotypes some degree of protection against bronchoconstriction remained after two weeks of β_2_-agonist use. After terbutaline pre-treatment, the differences in the dose response curves between genotypes were attenuated and the maximum difference in improvement in PD_20 _by the highest dose of fenoterol between the genotypes (II/III versus I/I, figure 1) was 1.5 doubling dose, where differences larger than about one doubling dose are generally considered to be clinically significant, in view of the confidence intervals for repeated determinations of methacholine bronchoprovocation thresholds [28]. It is evident that at lower doses of β_2_-agonist, such as normally used by asthmatics in a clinically stable state, the differences are even smaller. Altogether, this implicates that the functional phenotypical differences between the genotypes are probably only of limited clinical significance, at least in stable mild to moderate asthmatics as in our cohort. As we previously argued, such differences are likely to be more relevant in situations with a high state of functional antagonism, such as in asthma exacerbations with severe bronchoconstriction and functionally antagonized receptors by inflammatory mediators, when high doses of short acting β_2_-agonists are used [21].

## Conclusion

Our data and analyses in a cohort of asthmatic patients indicate differences between six distinct β_2_-AR allelic genotypes in the shape of the dose response relationship of a β_2_-AR agonist and in the propensity to develop tolerance for these effects. The genotypes are based upon combinations of three limited allelic haplotypes containing the functionally relevant parts of the β_2_-AR gene. The differences we found are statistically significant and functionally relevant only for bronchoprotection when compared to bronchodilation in terms of recovery from methacholine induced bronchoconstriction, and of a magnitude unlikely to be of clinical significance.

## Competing interests

The department of Pulmonology of the AMC (authors: AV, FRW, HMJ, REJ) received an unrestricted research grant for the conduction of this study and one additional study. There are no competing interests for the other authors.

## Authors' contributions

AV conducted the study, was involved in the analysis of the data and was involved in drafting the manuscript.

EAW aided in the carry out the molecular genetic assays and was involved in drafting the manuscript.

RW carried out the molecular genetic assays

FRW participated in the design of the study

GAMH performed the statistical analysis

HMJ participated in the design of the study and interpretation of the data

REJ participated in the design of the study and was involved in the analysis of the data and the drafting of the manuscript

## References

[B1] Green SA, Turki J, Innis M, Liggett SB (1994). Amino-terminal polymorphisms of the human beta 2-adrenergic receptor impart distinct agonist-promoted regulatory properties.. Biochemistry.

[B2] Green SA, Turki J, Bejarano P, Hall IP, Liggett SB (1995). Influence of beta 2-adrenergic receptor genotypes on signal transduction in human airway smooth muscle cells.. Am J Respir Cell Mol Biol.

[B3] Martinez FD, Graves PE, Baldini M, Solomon S, Erickson R (1997). Association between genetic polymorphisms of the beta2-adrenoceptor and response to albuterol in children with and without a history of wheezing.. J Clin Invest.

[B4] Lima JJ, Thomason DB, Mohamed MH, Eberle LV, Self TH, Johnson JA (1999). Impact of genetic polymorphisms of the beta2-adrenergic receptor on albuterol bronchodilator pharmacodynamics.. Clin Pharmacol Ther.

[B5] Israel E, Drazen JM, Liggett SB, Boushey HA, Cherniack RM, Cooper DM, Fahy JV, Fish JE, Ford JG, Kraft M, Kunselman S, Lazarus SC, Lemanske RF, Martin RJ, McLean DE, Peters SP, Sorkness CA, Szefler SJ, Weiss ST, Yandava CN, Chinchilli, VM, Silverman, EK (2000). The effect of polymorphisms of the beta(2)-adrenergic receptor on the response to regular use of albuterol in asthma.. Am J Respir Crit Care Med.

[B6] Israel E, Chinchilli VM, Ford JG, Boushey HA, Cherniack R, Craig TJ, Deykin A, Fagan JK, Fahy JV, Fish J, Kraft M, Kunselman SJ, Lazarus SC, Lemanske RFJ, Liggett SB, Martin RJ, Mitra N, Peters SP, Silverman E, Sorkness CA, Szefler SJ, Wechsler ME, Weiss ST, Drazen JM (2004). Use of regularly scheduled albuterol treatment in asthma: genotype-stratified, randomised, placebo-controlled cross-over trial. Lancet.

[B7] Hancox RJ, Sears MR, Taylor DR (1998). Polymorphism of the beta2-adrenoceptor and the response to long-term beta2-agonist therapy in asthma.. Eur Respir J.

[B8] Lipworth B, Koppelman GH, Wheatley AP, Le JI, Coutie W, Meurs H, Kauffman HF, Postma DS, Hall IP (2002). Beta2 adrenoceptor promoter polymorphisms: extended haplotypes and functional effects in peripheral blood mononuclear cells. Thorax.

[B9] Jackson CM, Lipworth B (2004). Benefit-risk assessment of long-acting beta2-agonists in asthma. Drug Saf.

[B10] Tan S, Hall IP, Dewar J, Dow E, Lipworth B (1997). Association between beta 2-adrenoceptor polymorphism and susceptibility to bronchodilator desensitisation in moderately severe stable asthmatics.. Lancet.

[B11] Taylor DR, Hancox RJ, McRae W, Cowan JO, Flannery EM, McLachlan CR, Herbison GP (2000). The influence of polymorphism at position 16 of the beta2-adrenoceptor on the development of tolerance to beta-agonist. J Asthma.

[B12] Lipworth BJ, Hall IP, Aziz I, Tan KS, Wheatley A (1999). Beta2-adrenoceptor polymorphism and bronchoprotective sensitivity with regular short- and long-acting beta2-agonist therapy.. Clin Sci (Colch ).

[B13] Lee DK, Jackson CM, Bates CE, Lipworth BJ (2004). Cross tolerance to salbutamol occurs independently of beta2 adrenoceptor genotype-16 in asthmatic patients receiving regular formoterol or salmeterol. Thorax.

[B14] Chong LK, Chowdry J, Ghahramani P, Peachell PT (2000). Influence of genetic polymorphisms in the beta2-adrenoceptor on desensitization in human lung mast cells.. Pharmacogenetics.

[B15] Moore PE, Laporte JD, Abraham JH, Schwartzman IN, Yandava CN, Silverman ES, Drazen JM, Wand MP, Panettieri RAJ, Shore SA (2000). Polymorphism of the beta(2)-adrenergic receptor gene and desensitization in human airway smooth muscle. Am J Respir Crit Care Med.

[B16] McGraw DW, Forbes SL, Kramer LA, Liggett SB (1998). Polymorphisms of the 5' leader cistron of the human beta2-adrenergic receptor regulate receptor expression.. J Clin Invest.

[B17] Parola AL, Kobilka BK (1994). The peptide product of a 5' leader cistron in the beta 2 adrenergic receptor mRNA inhibits receptor synthesis.. J Biol Chem.

[B18] Scott MG, Swan C, Wheatley AP, Hall IP (1999). Identification of novel polymorphisms within the promoter region of the human beta2 adrenergic receptor gene.. Br J Pharmacol.

[B19] Drysdale CM, McGraw DW, Stack CB, Stephens JC, Judson RS, Arnold K, Ruano G, Liggett SB, Nandabalan (2000). Complex promoter and coding region beta 2-adrenergic receptor haplotypes alter receptor expression and predict in vivo responsiveness.. Proc Natl Acad Sci U S A.

[B20] Westland R, van Veen A, Jansen HM, Jonkers RE, Wierenga EA (2004). Limited impact of multiple 5' single-nucleotide polymorphisms on the transcriptional control of the human beta 2-adrenoceptor gene. Immunogenetics.

[B21] van Veen A, Weller FR, Wierenga EA, Jansen HM, Jonkers RE (2003). A comparison of salmeterol and formoterol in attenuating airway responses to short-acting beta2-agonists. Pulm Pharmacol Ther.

[B22] McGraw DW, Almoosa KF, Paul RJ, Kobilka BK, Liggett SB (2003). Antithetic regulation by beta-adrenergic receptors of Gq receptor signaling via phospholipase C underlies the airway beta-agonist paradox. J Clin Invest.

[B23] Lipworth B, Tan S, Devlin M, Aiken T, Baker R, Hendrick D (1998). Effects of treatment with formoterol on bronchoprotection against methacholine.. Am J Med.

[B24] Scola AM, Chong LK, Chess-Williams R, Peachell PT (2004). Influence of agonist intrinsic activity on the desensitisation of beta2-adrenoceptor-mediated responses in mast cells. Br J Pharmacol.

[B25] Molimard M, Naline E, Zhang Y, Le GV, Begaud B, Advenier C (1998). Long- and short-acting beta2 adrenoceptor agonists: interactions in human contracted bronchi.. Eur Respir J.

[B26] Palmqvist M, Ibsen T, Mellen A, Lotvall J (1999). Comparison of the relative efficacy of formoterol and salmeterol in asthmatic patients.. Am J Respir Crit Care Med.

[B27] Wraight JM, Hancox RJ, Herbison GP, Cowan JO, Flannery EM, Taylor DR (2003). Bronchodilator tolerance: the impact of increasing bronchoconstriction. Eur Respir J.

[B28] Crapo RO, Casaburi R, Coates AL, Enright PL, Hankinson JL, Irvin CG, MacIntyre NR, McKay RT, Wanger JS, Anderson SD, Cockcroft DW, Fish JE, Sterk PJ (2000). Guidelines for methacholine and exercise challenge testing-1999. This official statement of the American Thoracic Society was adopted by the ATS Board of Directors, July 1999. Am J Respir Crit Care Med.

